# Robotic-assisted resection of parametrial endometriosis with ureterolysis of a medially distorted ureter using indocyanine green and near-infrared fluorescence

**DOI:** 10.1093/jscr/rjaf1064

**Published:** 2026-01-08

**Authors:** Philippe Van Trappen, Gaby Moawad, Christophe Ghysel, Harm Arentsen

**Affiliations:** Department of Gynecology and Gynecological Oncology, AZ Sint-Jan Hospital Bruges, Ruddershove 10, 8000 Bruges, Belgium; Department of Obstetrics and Gynecology, The George Washington University Hospital, 900 23rd St Nw, Washington, DC 20037-2342, United States; Department of Urology, AZ Sint-Jan Hospital Bruges, Ruddershove 10, 8000 Bruges, Belgium; Department of Urology, AZ Sint-Jan Hospital Bruges, Ruddershove 10, 8000 Bruges, Belgium

**Keywords:** endometriosis, ICG, infrared fluorescence, parametrium, robotic, ureter

## Abstract

Deep infiltrating endometriosis (DIE) is a severe form of endometriosis, with endometriotic implants that can invade several anatomical structures. The urinary tract is the second most common site for deep endometriosis with ureteral endometriosis (UE) the most challenging to manage. Multidisciplinary surgery along with hormonal therapy is the corner stone for the treatment of DIE. Here we describe, with a video, a surgical technique using robotic-assisted surgery along with retrograde injection of indocyanine green (ICG) in the ureter for resection of parametrial endometriosis and ureterolysis using near-infrared fluorescence. The case was a 29-year-old nullipara with a history of severe dysmenorrhea and chronic pelvic pain. The patient was discharged after 48 hrs. The advantages of robotic surgery and the use of ICG in the ureter, in selected cases with UE, enables careful ureterolysis as ICG can even be absorbed in the ureteral mucosa/wall when no stent is left behind during surgery.

## Introduction

Endometriosis is a chronic medical condition, affecting approximately 10% of women in their reproductive age, with the extra-uterine presence of ectopic implants of endometrial-like glands/stromal tissue [1]. The urinary tract represents the second most common site for deep infiltrating endometriosis (DIE) with ureteral endometriosis (UE) having a prevalence up to 23%, affecting mostly the distal ureter in the parametrium [2]. UE can be subdivided into intrinsic and extrinsic forms. Intrinsic UE involves endometriosis and/or fibrosis in either the muscular or uroepithelial layer, whereas extrinsic UE is characterized by the endometriotic invasion of the ureteric adventitia or more often the nearby connective tissue of the parametrium, which may lead to compression or distortion of the ureter [3]. Because of the often difficult challenge to perform successfully ureterolysis in case of extensive DIE in the pelvis, indocyanine green (ICG) was introduced to visualize the ureter with this fluorescent tracer in near-infrared (NIR) light [1, 4–6].

## Case report

**Figure 1:**
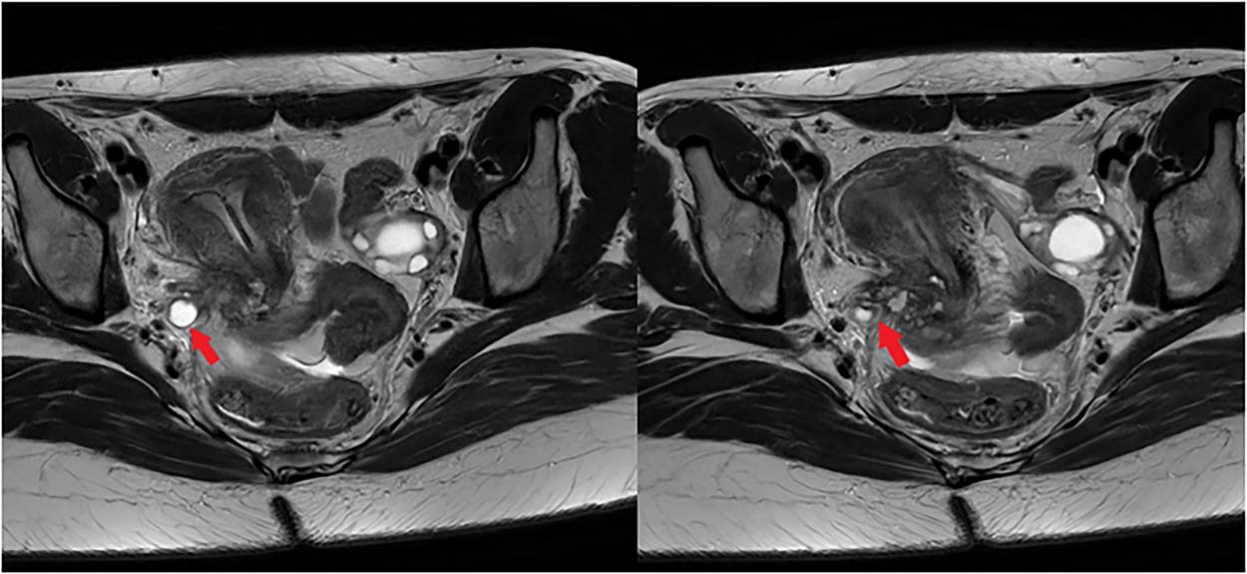
The left image illustrates a dilated right ureter (arrow) due to the presence of endometriosis in the right parametrium, as a T2-hypointense cordly-shaped zone in the parametrium. The right image shows endometriosis in the right antero-lateral parametrium encasing (arrow) the right distal ureter.

A 29-year-old G1P0A1 with a history of severe dysmenorrhea and chronic pelvic pain was referred to our tertiary center for surgery, after being diagnosed with DIE in the right parametrium and encasing the right ureter on ultrasonography. Further preoperative imaging with magnetic resonance imaging (MRI) revealed DIE on the posterior wall of the cervix with several T2 hypo-intense small cysts (with protein rich content), extending to the torus uterinus and to thickened right uterosacral ligament; the right ureter was dilated (hydroureter) and medially distorted by a T2-hypointense cordly-shaped zone in the right parametrium (Fig. 1). The endometriosis in the right antero-lateral parametrium was encasing the right distal ureter (Fig. 1); there was evidence of hydroureteronephrosis.

At our multidisciplinary endometriosis team meeting the proposed surgical procedure was a robotic-assisted resection of the parametrial endometriosis and endometriosis posteriorly to the cervix, with complete ureterolysis using ICG in the ureter and Firefly technology at the robotic console. The da Vinci Xi robot platform (Intuitive Surgical Inc.) was used with the 4 robotic arms, with dual console (gynecologist/urologist). Prior to the surgical procedure 5 cc of ICG (2 mg/mL) was injected retrogradely in the ureter with a straight whistle-tip ureteral catheter (Supplementary Video; https://youtu.be/oz2k_S9L_Vk), without leaving a temporary ureteral stent in place during the surgery.

**Figure 2:**
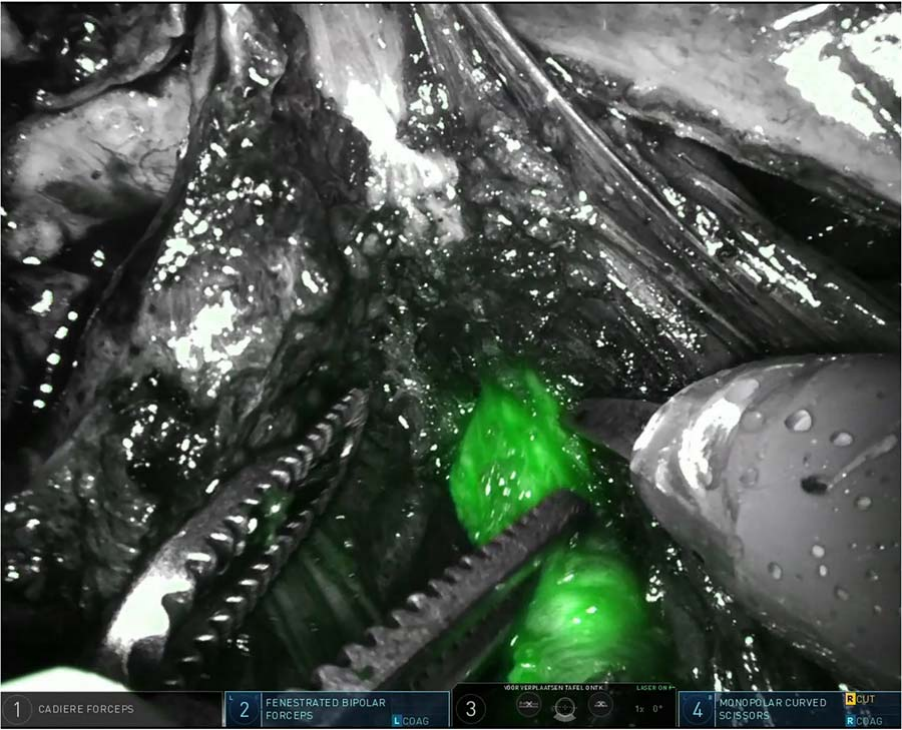
Easy identification of the surgical margins between the connective tissue in the parametrium and the distal ureter using ICG.

**Figure 3:**
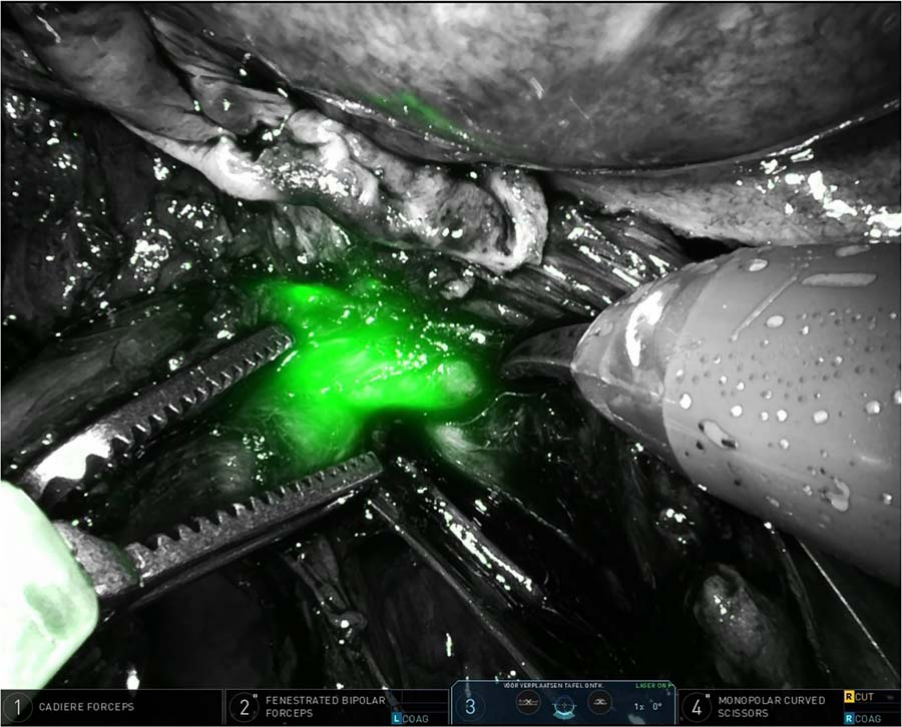
Partially stenosed and medially distorted ureter visualized by ICG and NIRF.

The right pelvic side wall was opened to identify and mobilize the right stenosed ureter along its course in the right pelvic side wall, towards its crossing with the uterine artery. Using intermittent near-infrared fluorescence (NIRF) imaging we could easily identify the right distal ureter, highlighted by ICG (Fig. 2). Ureterolysis was facilitated by the ICG absorption in the ureteral mucosa/wall as visible during the procedure (Supplementary Video; https://youtu.be/oz2k_S9L_Vk). Careful dissection of the fibrotic tissue around the stenosed ureter resulted in release of the ureter and the area where the partial stenosis was present (Fig. 3). The parametrial endometriosis was further dissected and mobilized towards the posterior cervix. Endometriosis was apparently invading the posterior lip of the cervix with several small cysts/cavities filled with dark brown ‘chocolate-like’ endometrial fluid, making clear margins difficult for resection (Supplementary Video; https://youtu.be/oz2k_S9L_Vk). All the cysts/cavities in the posterior cervix were resected and the superficial cervical stroma was coagulated with spray diathermy. The whole endometriosis area was further dissected towards the posterior vaginal wall, with opening the vagina in order to achieve complete resection of the deep endometriosis. At the end of the procedure a prophylactic double-J stent was placed in the right ureter for 3 months to prevent secondary fibrosis/stenosis. The patient was discharged after 48 hrs. The renal function postoperatively was normal. As hormonal therapy she received relugolix combination therapy (relugolix 40 mg, estradiol 1 mg, norethisterone acetate 0·5 mg). An MRI scan four months after surgery showed no evidence of residual or recurrent deep infiltrative endometriosis (Supplementary Video; https://youtu.be/oz2k_S9L_Vk). A clinical visit five and ten months after surgery revealed no significant pain complaints, with normal daily activities. An expert ultrasound scan 10 months after surgery revealed no evidence of recurrent deep endometriosis.

## Discussion

Here we describe a case of DIE in a young patient, with endometriosis invading the posterior cervix and extending into the posterior vaginal wall, the right uterosacral ligament and the right antero-lateral parametrium, encasing the distal right ureter. A robotic-assisted resection of all the DIE was performed, with careful ureterolysis using retrograde injection of ICG in the ureters and NIRF imaging at the robotic console, without leaving a ureteral stent during the surgical procedure.

Parametrial endometriosis has an incidence of 0.1% to 1% in patients with endometriosis and constitutes a complex manifestation of DIE, where endometriotic nodules can cause extrinsic compression on the distal ureter due to invasion or extensive fibrosis in the peri-ureteral connective tissue, which can lead to severe hydronephrosis [3]. The presence of extensive fibrosis in the connective tissue, due to endometriosis, surrounding the distal ureter constitutes a surgical challenge for performing a successful ureterolysis. In the case presented, careful ureterolysis was performed using intermittent NIRF-ICG imaging for visualizing the ureter and mobilizing the partially stenosed and medially distorted ureter along its dissection (Supplementary Video; https://youtu.be/oz2k_S9L_Vk). After isolating and mobilizing the right ureter, an antero-lateral parametrectomy was performed with preservation of the posterolateral parametrium including the lower hypogastric nerves and the inferior hypogastric plexus.

In stage IV endometriosis, Thigpen *et al*. [5] demonstrated in 2022 a similar approach during a robotic-assisted hysterectomy with bilateral ureterolysis and low anterior bowel resection, but in their case ICG was injected into each ureteral catheter with the catheters left in place during the surgical procedure, and with caps on the catheters to maximize ICG retention. In the case presented, ICG was injected retrogradely in the partially stenosed and medially displaced ureter using a straight whistle-tip ureteral catheter, without leaving a catheter in the ureters. With this particular procedure we could observe ICG absorption in the ureteral mucosa/wall during the whole surgical procedure, and with the advantage of having a flexible ureter during the dissection. Guan *et al*. [6] described in 2023 a robotic-assisted transvaginal natural orifice transluminal endoscopic surgery (vNOTES) hysterectomy with resection of DIE, using 5 cc of ICG injected in the ureters after insertion of bilateral ureteral stents, which remained during the surgical procedure. Besides using ICG in the ureter, ICG can also be applied intravenously for visualizing the vascular perfusion of the ureteral wall or at bowel anastomosis [1, 7–9].

In conclusion, in case no ureteral stent is left behind during the surgical procedure for UE as demonstrated here in the video, ICG can be absorbed in the ureteral mucosa/wall and provides clear visibility during the ureterolysis using NIR imaging.
